# Mental health impact and mental health care among first responders following the Paris terror attacks in November 2015

**DOI:** 10.1093/eurpub/ckac129.337

**Published:** 2022-10-25

**Authors:** Y Motreff, P Pirard, C Vuillermoz, G Rabet, M Petitclerc, L Stene, T Baubet, S Vandentorren

**Affiliations:** 1 Santé Publique France, French National Public Health Agency, Saint-Maurice, France; 2 Inserm, Department of Social Epidemiology, Paris, France; 3 Departement of Psychology, Université Paris, Paris, France; 4 NKVTS, Norwegian Centre for Violence and Traumatic Stress Studies, Oslo, Norway

## Abstract

**Introduction:**

During the evening of 13 November 2015, a terror attack occurred in France in the Paris area. Overall, 130 people were killed, 643 were injured and several thousands were psychologically impacted. Thousands of first responders (FRs), including health professionals, firefighters, affiliated volunteers and police officers were mobilized that night and during the subsequent weeks. The aims of our study were to measure the psychological impact on FRs, and its associated factors 12 months after the 13 November 2015 terrorist attacks, as well as their engagement in mental health care and its associated factors.

**Methods:**

FRs who had intervened during the night and/or the aftermath of the terror attacks had the possibility of answering a web-based study 8-12 months after the attacks. They satisfied criterion A of the DSM 5 definition of Post Traumatic Stress Disorder (PTSD). PTSD and partial PTSD were measured using the PTSD checklist for DSM-5 (PCL-5) and depression with the hospital anxiety and depression (HAD) scale.

**Results:**

Overall, 663 FRs were included in the analysis. Prevalence of PTSD in our sample went from 3.4% among firefighters to 9.5% among police officers. Low educational level, social isolation, intervention on unsecured crime scenes and lack of training were associated with PTSD. Among FRs with PTSD, partial PTSD or depression, 38% sought mental health care. Mental health care engagement was associated with a history of mental health care, post-immediate support and the presence of PTSD, partial PTSD or depression.

**Conclusions:**

Our results highlight that improving access to mental health care for FRs after terror attacks is needed. Efforts should be made before and after potentially traumatic events to ensure mental health education for FRs. Special attention should be given to FRs living in social isolation, those with low educational levels and those who intervene in unsecured crime scenes.

